# A Comparative Study of Two *Oroxylum indicum* (L.) Kurz. Phenotypes Based on Phytochemicals and Antioxidant Effects, and the Anti-Inflammatory Activity of Leaf and Pod Extracts

**DOI:** 10.3390/plants13152110

**Published:** 2024-07-30

**Authors:** Pattaraphorn Panomai, Suthasinee Thapphasaraphong, Natsajee Nualkaew

**Affiliations:** Faculty of Pharmaceutical Sciences, Khon Kaen University, Khon Kaen 40002, Thailand; pattar@kkumail.com (P.P.); sutpit1@kku.ac.th (S.T.)

**Keywords:** *Oroxylum indicum*, phenotype, antioxidants, anti-inflammation, principal component analysis, HPLC method validation

## Abstract

Indian trumpet tree *Oroxylum indicum* (L.) Kurz exhibits a wide range of biological activities in all plant parts, including anti-inflammation, antioxidant, and wound-healing activities. In Thailand, there are tall- and short-stem phenotypes. The latter are preferred for commercial cultivation due to their fast growth and lower harvesting cost. This study aimed to compare the chemical profiles and antioxidant effects of leaves and young pods between two phenotypes using principal component analysis (PCA) and then to evaluate the anti-inflammatory potential of the selected phenotype’s plant parts. The biomarker contents were quantified by HPLC. The antioxidants were determined using the DPPH, ABTS, and FRAP models. Nitric oxide (NO) production assays in LPS-induced RAW264.7 macrophages were performed to determine the anti-inflammatory property of the extracts. The PCA revealed that there were no differences in total phenolic content, total flavonoid content, or antioxidant activities between short- and tall-stem phenotypes. Higher potency of the NO-inhibitory effect was achieved from the leaf extract than the pod extract. These results support using the short-stem phenotypes for utilizing the leaf and pod of *O. indicum*, and suggest choosing the leaf part for further anti-inflammatory product development.

## 1. Introduction

Indian trumpet tree *Oroxylum indicum* (L.) Kurz. (Bignoniaceae) is a plant native to India and exhibits an extensive distribution across Southeast Asia, including Laos, Vietnam, Malaysia, Indonesia, and Thailand [1]. Its plant parts have been used as traditional medicine in various countries. In Thailand, the root is used for anti-fever treatment, the seed is used as anti-tussive treatment, and the roasted immature pod is consumed as a local vegetable. The stem bark is used for the treatment of arthritis and for wound healing [2] and is a constituent of some herbal recipes, i.e., *ya-pha-sa-kan-phu*, *ya-lueang-pid-sa-mhud*, and *ya-luead-ngam*, which are entered on the list of Herbal Medicinal Products of the National List of Essential Medicines (NLEMs) [3]. The major bioactive chemicals of this plant are flavones, such as baicalin, baicalein, chrysin, and oroxylin A. Interestingly, those substances are also involved in Chinese herbal medicine using *Scutellaria baicalensis*, which demonstrates similar pharmacological activities to those of *O. indicum*, such as antiviral, antioxidant, and anti-inflammatory activities [4].

**Figure 1:**
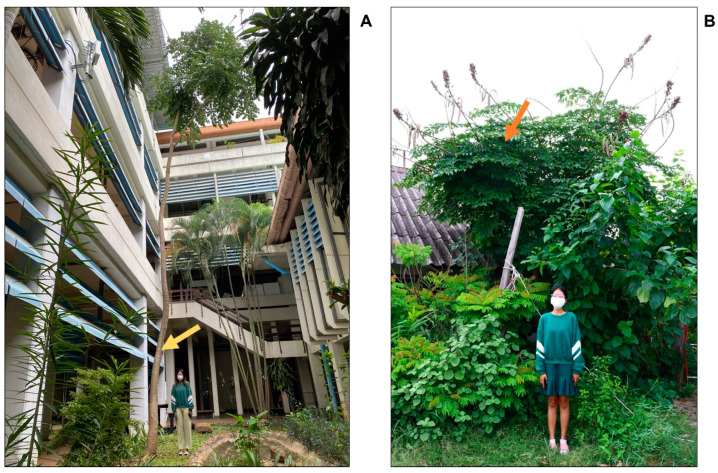
Indian trumpet tree *Oroxylum indicum.* (**A**) A tall-stem phenotype in Khon Kaen province; (**B**) a short-stem phenotype in Khon Kaen province; the arrows point to this plant.

*O. indicum* grows naturally to 5–20 m (Figure 1A) in dry mixed forest and dry evergreen forest. In India, the species has a wide distribution in moist hill forests. Due to the low rate of seed germination and habitat destruction, along with the changing climate, this plant is considered threatened in India [5]. The tall stem grown naturally of this plant leads to a difficult harvest for use as medicine and food. In Thailand, the short-stem type (Figure 1B) occurs through natural mutation, and root cutting has been widely propagated. This phenotype is conserved and supports rapid commercial distribution through asexual reproduction methods such as root cutting and stem cutting. This has allowed *O. indicum* to become an economically important plant for health benefits such as food supplements and herbal medicines (the seeds) and as a vegetable (the immature pod) available in local markets in northeastern Thailand, while the leaves are less utilized.

Natural variations in plants arise from various factors, including mutations in genes controlling stem height and environmental influences. These variations affect physical appearances, fruiting times, and chemical compositions, which impact the potency of biological activities. The tall- and short-stem phenotypes of *O. indicum* are different in fruiting period and stem height. The short stem (strains Phet None Phueng, and Bao Dor) variety is approximately 5 m in height and polycarpic with the flowering period. It generates rapid growth, a lot of flowers and fruit, and has a long fruit duration throughout the year. On the other hand, the tall-stem variety grows up to 20 m in height, is monocarpic, and has a flowering period from March to July. Although the leaflets of both types have the same appearance, their pods differ in morphology. The immature pod from the tall type is longer in shape with a light-green color, whereas those of the short stems are shorter and have a greenish violet color, as shown in Table 1.

The propagation method, such as root cutting, might change the root system of regenerated plants, such as *Cunninghamia lanceolata*, which in turn affects the morphology and characteristics of the aerial part [6]. Various factors influencing plant height may lead to differences in phytochemical levels between the two *O. indicum* phenotypes and have consequences for their biological potency. To justify the use of the short-stem type as a source of material supply for health product manufacturing, this study was designed to compare the tall- and short-stem types of *O. indicum* based on the correlation of the phytochemicals and antioxidant activities using principal component analysis (PCA).

PCA has become a popular method in several research areas. It provides correlations between parameters. In this study, the tall- and short-stem phenotypes were evaluated for chemical profiles and antioxidant properties, which led to the selection of a suitable phenotype. The phytochemical content of the plant parts was grouped by a PCA score plot. After that, the plant part selection for further anti-inflammatory product development was considered between leaf and pod extracts of the selected phenotypes by nitric oxide-inhibitory activity assays.

## 2. Results

**Figure 2:**
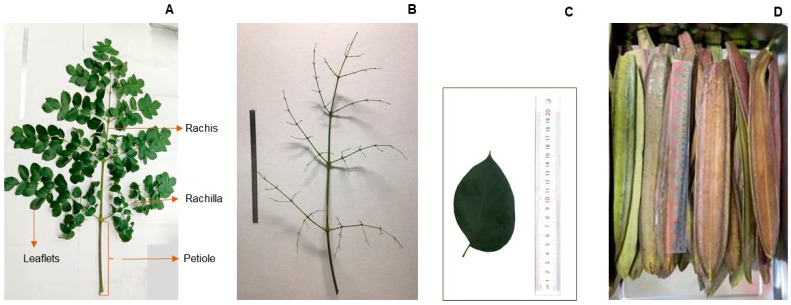
Parts of *O. indicum* leaves and pods used in this study. (**A**) The whole 3-pinnate compound leaf (PL); (**B**) petiole, rachis, and rachilla (P); (**C**) leaflet (L); (**D**) immature pods (Pds).

The leaves and the immature pods of the tall- and short-stem phenotypes were compared for phytochemicals and antioxidant properties. Since the leaves of Indian trumpet tree *O. indicum* are very large, they were separated into the leaflet (L); petiole, rachis, and rachilla (P); and whole leaves (PL), as shown in Figure 2, to determine beneficial parts for use.

### 2.1. Extraction and Characterization of the Extracts

The yields from 80% ethanol extraction, total phenolic contents (TPCs), and total flavonoid contents (TFCs) of the individual plants are shown in Table S1, which demonstrated the independent differences among each source.

A comparison between the short- and tall-stem phenotypes showed no statistical differences in terms of percentage yields, TPCs, or TFCs, as shown in Table 2. All extracts contained higher TPCs than TFCs, which suggested the presence of various types of phenolic compounds besides flavonoids. The leaflet extract (LE) had TPCs and TFCs similar to those of the whole leaf (PLE), while those of the petiole, rachis, and rachilla extract (PE) were the lowest. These results indicated the presence of phenolic compounds and flavonoids in all parts of the leaf, and suggested that the whole leaf might be more beneficial rather than separating it into the leaflet or petiole.

Pod extracts (PdEs) from both phenotypes gave higher percentage yields and lower TPCs and TFCs than the leaves (PE, LE, and PLE), which indicated high amounts of the other groups available in the PdEs, probably primary metabolites such as lipids, fatty acids, amino acids, and sugars, which are generally stored in the fruits and can be extracted using 80% EtOH.

### 2.2. HPLC Profile of O. indicum Extracts

**Figure 3:**
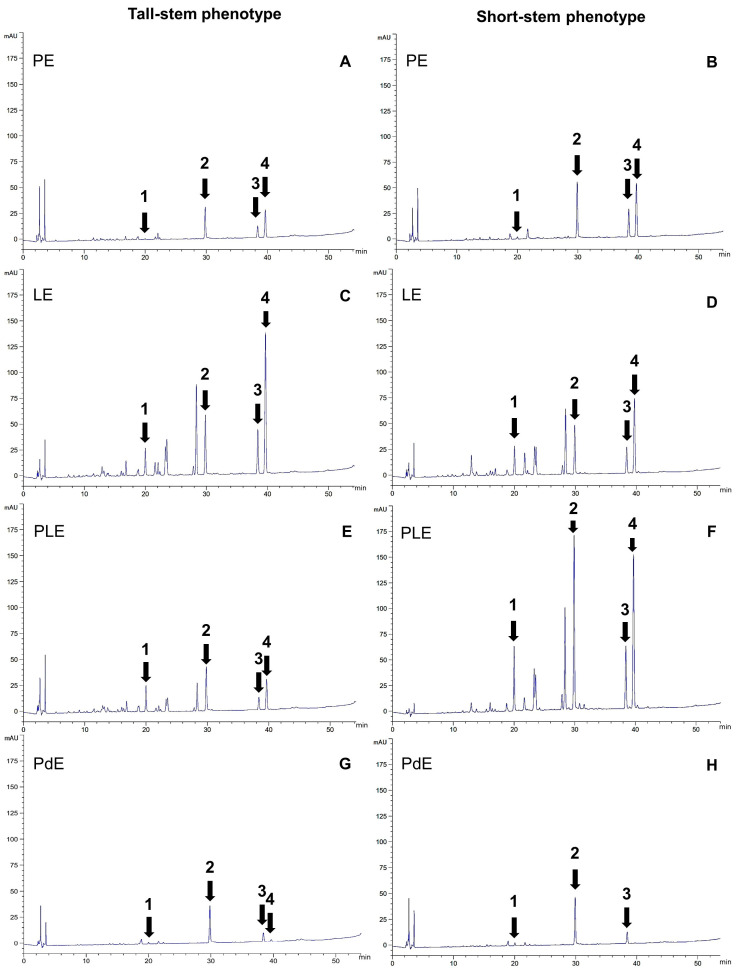
HPLC chromatograms of the leaves and pod extracts (1 mg/mL of 10 µL) of *O. indicum* from Buengkarn province: (**A**) rachis and petiole extract (PE) of a tall-stem phenotype; (**B**) PE of a short-stem phenotype; (**C**) leaflet extract (LE) of a tall-stem phenotype; (**D**) LE of a short-stem phenotype; (**E**) whole-leaf extract (PLE) of a tall-stem phenotype; (**F**) PLE of a short-stem phenotype; (**G**) pod extract (PdE) of a tall-stem phenotype; (**H**) PdE of a short-stem phenotype. Peak 1: baicalin; peak 2: baicalein; peak 3: chrysin; and peak 4: oroxylin A.

The HPLC chromatograms of the leaf and pod extracts displayed similar patterns between the tall- and short-stem phenotypes, but different peak heights, as shown in Figure 3. The HPLC pattern of PLE was a combination of LE and PE. This result clearly supports the same chemical profile of the tall- and short-stem phenotypes of *O. indicum*.

### 2.3. Quantitative Analysis of Biomarkers in O. indicum Extracts

Since there has been no previous report of an HPLC method for the quantitative analysis of biomarker contents of baicalin, baicalein, chrysin, and oroxylin A in the same HPLC chromatogram, an HPLC method was developed and hence needed to be validated before use. The results of validation were according to AOAC guidelines [7] are shown in Table S2.

The average biomarker contents of the tall- and short-stem phenotypes (Table 3) demonstrated that the leaves contained mainly baicalin, baicalein, and oroxylin A, which was consistent with the amounts found in each individual source (Table S2). The total amount of biomarkers in the whole-leaf extracts (PLEs) was higher than that in the PdEs, and the total biomarker amount of PLEs was higher in the short-stem phenotype than in the tall-stem phenotype.

### 2.4. Antioxidant Activities of O. indicum Extracts

**Figure 4:**
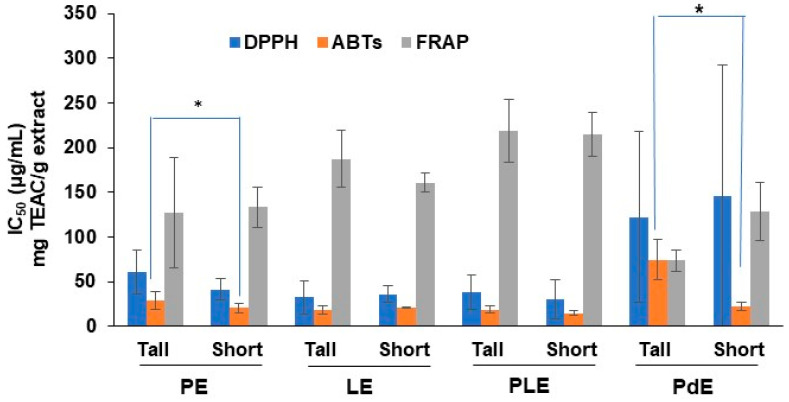
Antioxidant activities of leaf extracts (PE, LE, PLE) and pod extracts (PdEs) of the tall- and short-stem phenotypes. Data are expressed as the mean ± standard error of the mean (SEM). The IC_50_ values of Trolox are 5.91 ± 0.02, and 4.18 ± 0.05 µg/mL from the DPPH and ABTS assays, respectively. * *p* < 0.05 between the tall- and short-stem phenotypes.

The antioxidant effects of *O. indicum* extracts from tall- and short-stem phenotypes were of moderate potency. PdEs’ effects were less than those of leaf extracts (PE, LE, and PLE), as shown in Figure 4, which was in line with its lower TPCs and TFCs (Table 2). There were no significant differences in the antioxidant properties found in the DPPH and FRAP assays between the leaf extracts of the tall- and short-stem phenotypes (*p* < 0.05), but the results of the ABTS model revealed statistically significant differences in PEs and PdEs between the two phenotypes.

### 2.5. Principal Component Analysis (PCA) of Two Phenotypes

The PCA score plots demonstrated the distribution of data for nine parameters of the phytochemical contents (TPCs; TFCs; baicalin, baicalein, chrysin, and oroxylin A; the IC_50_ from the DPPH; the ABTS; and the FRAP values of the extracts). Each dot in the plots represents each location of *O. indicum* (a total of six locations involved the tall- and short-stem phenotypes). Those values from each plant are shown in Tables S1 and S3.

**Figure 5:**
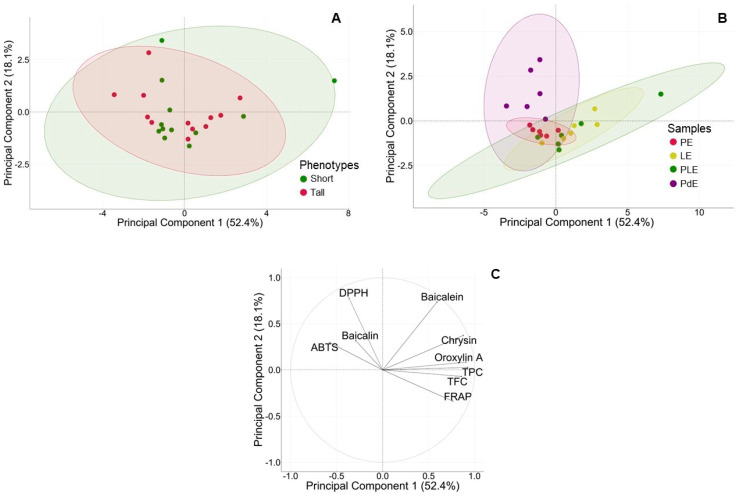
Principal component analysis (PCA) score plot of *O. indicum* extracts (PE, LE, PLE, and PdE) collected from 6 locations. (**A**) The average values of nine phytochemical parameters of the tall− and short−stem phenotypes were plotted and demonstrated no difference between groups (green spots: short-stem phenotype; red spots: tall-stem phenotype). (**B**) The average values of phytochemical contents of the extracts from each part of *O. indicum* (PE, LE, PLE, and PdE) are plotted. Separation of data into 2 groups was observed. The leaf group (PE, LE, and PLE) was in the tilted right oval, and the pod (PdE) was in the upright oval. Red spots: rachis, rachilla, and petioles (PEs); yellow spots: leaflets (LEs); green spots: whole leaves (PLEs); and purple spots: pods (PdEs). (**C**) Loading plot of *O. indicum* phytochemical data (each parameter was judged from 6 samples, which comprised 3 tall–stem and 3 short–stem phenotypes).

The two-dimensional plots consisted of the principal components 1 and 2 (PC1 and PC2), which accounted for 52.4% and 18.1% of the variance, respectively. The plots showed 70.5% of the total variance of the data and were clustered based on two factors, the phenotypes (tall and short) and the plant parts (P, L, PL, and Pd), as shown in Figure 5A,B, respectively.

The PCA plot showed that all nine parameters were the same for the tall- and short-stem phenotypes, as shown by the complete overlapping of the short phenotype (green ellipse) and tall phenotype (red ellipse), illustrated with 95% confidence (Figure 5A). This result indicated that the tall- and short-stem types were the same. Thus, the selection of the suitable source of raw material could not be made from the stem height phenotype.

Regarding the separated parts of the *O. indicum* leaf, the overlapping of the three eclipses for the PE (red ellipse), LE (yellow ellipse), and PLE (green ellipse) revealed that they were not significantly different between the extracts from either the separated parts or the whole leaf. Partial overlapping of the PdE (purple ellipse) with the leaf parts (PE, LE, and PLE) was observed (Figure 5B) and suggested differences between the leaf and the pod.

Since the stem height phenotype of *O. indicum* did not affect the chemical content or antioxidant activities, based on the lower cost of its harvesting process, the short phenotype was selected for further studies.

The correlation between phytochemicals, biomarker contents, and antioxidant activities was analyzed using a loading plot, as shown in Figure 5C. There were two principal components, PC1 and PC2. PC1 included TPC, oroxylin A, chrysin, TFC, and FRAP, while PC2 consisted of DPPH, ABTS, baicalein, and baicalin. These plots suggested the relationships among those parameters. TPC had a strong relationship with TFC, which was in line with the data shown in Table 2. Both TPC and TFC also had positive relations with oroxylin A, chrysin, and FRAP, which exhibited acute angles with each other and resulted in a contribution to PC1. DPPH had a positive relationship with ABTS, as reflected by the acute angle between them. In addition, DPPH and ABTS had negative relationships with TPC, TFC, oroxylin A, chrysin, and FRAP, as indicated by the obtuse angles among these parameters.

### 2.6. Anti-Inflammatory Activities of S-BK Extracts in LPS-Induced RAW 264.7 Macrophages

PCA demonstrated that the tall- and short-stem phenotypes of *O. indicum* did not differ. Therefore, the short stem was chosen for further evaluating the anti-inflammatory effect of the leaf and pod extracts. Since the short-stem phenotype from Buengkarn province (S-BK) had the highest level of overall biomarkers among the short-stem phenotypes (Table S1), it was chosen to perform an assay. The inhibition of NO production in LPS-induced RAW 264.7 macrophages was used to evaluate the anti-inflammatory property of the extracts from *O. indicum* S-BK.

LPS treatment significantly increased NO production (*p* < 0.05) in RAW 264.7 macrophages, and NO production was ameliorated by PE, LE, PLE, and PdE in a dose-dependent manner at non-cytotoxic concentrations, as shown in Figure S1. IC_50_ values of 235.33, 57.61, 77.58, and 303.01 μg/mL, respectively, were obtained (Table 4). The potency of LE and PLE was comparable, and both were stronger than PE and PdE. Therefore, considering the zero-waste concept, PLE was chosen for further development into an anti-inflammatory product.

The anti-inflammatory effect of the biomarkers baicalin, baicalein, chrysin, and oroxylin A were also determined (Figure S1), and we found IC_50_ values of 5.86, 7.31, >400, and 10.46 μg/mL, respectively (Table 4).

## 3. Discussion

Plant height is influenced by both genetic and environmental factors. The advantages of short-stem plants include a greater yield from the mechanical harvest process and resistance to lodging, and those advantages promote plant breeding of shorter varieties [8,9]. A gene associated with a shortened stem has also been manipulated for the production of short phenotypes. Environmental conditions such as the light intensity, photoperiod, temperature, and soil properties [9] affect metabolism in plant cells, which results in different levels of secondary metabolite production, potency of biological activities, and plant phenotypes [10].

### 3.1. Extraction Yield and the Chemical Constituents of the Extracts

Indian trumpet tree *O. indicum* has two or three large pinnate compound leaves of approximately 1 m in length. The thick petioles and many rachides and rachillae provide a large biomass of cell walls and fiber, which influence the weight of the whole leaf, the extraction yield, the chemical contents, and the antioxidant properties. Moreover, the petioles, rachides, and rachillae may contain phenolic compounds bound to cellulose or plant fiber [11,12]. Therefore, the whole leaf (PL) and its separated parts (the petiole, rachis, and rachilla (P), and leaflets (L)) were evaluated in terms of their chemical and biological profiles.

Higher extraction yields were achieved from pods compared to leaves. PdE exhibited a lower total phenolic content (TPC) and total flavonoid content (TFC), indicating the presence of other phytochemicals besides phenolics that were extracted by 80% ethanol. Immature *O. indicum* pods are known to contain high levels of crude carbohydrate (67.5% dry weight) and crude protein (8.45% dry weight) [13], which could have contributed to the composition of PdE in this study.

The flavonoid contents were higher in the leaf extracts (PE, LE, and PLE) than the immature pod extracts (PdEs). Leaves contained baicalin, baicalein, chrysin, and oroxylin A, whereas the dominant biomarkers in PdE were baicalin and baicalein. These results were in line with those of Rojsanga et al. [14], who found that baicalin is the major compound of fruit and seed extracts (0.4–11% *w/w*) and that all flavones are present in low amounts in young fruit extracts (<1% *w/w* of the extract). Ethanolic extract from fruit collected from Nakhon Pathom and Chiang Rai province contained baicalin and baicalein as its major flavonoids and trace amounts of chrysin [15]. The available HPLC methods of *O. indicum* extracts include the separation of baicalin and baicalein [16]; baicalein and chrysin [17]; and baicalein, chrysin, and oroxylin A [18], but not the detection of baicalin, baicalein, chrysin, or oroxylin A in the same HPLC chromatogram. Therefore, the HPLC method was developed and validated in this study.

*O. indicum* is a plant of low genetic diversity, which leads to its difficulty in adapting to environmental variations [19]. The short-stem phenotype in Thailand has arisen from natural selective mutants and root-cutting propagation. The similar chemical profiles in this study supported the notion that both phenotypes of this plant belong to the same species. Therefore, their different amounts of bioactive compounds may be a result of influence from the habitat. Generally, root- and stem-cutting propagation methods are used to achieve the bulk-scale distribution of plants and can conserve plant phenotypes. However, these methods may affect the root system [20], and growth environments can change the catalytic activation of the secondary metabolites’ biosynthetic enzymes [21]. Both are the principal causes of the various levels of bioactive compounds, which impact the biological activities of the plant extracts.

### 3.2. The Relationship between Biomarker Contents and Antioxidant Activities

In previous studies, the IC_50_ values from DPPH assays of baicalin, baicalein, chrysin, and oroxylin A have been reported to be 17–43.2, 37.18, 42.7, and >100 µg/mL, respectively [22,23,24]. These demonstrated the moderate antioxidant potency of the biomarkers, which was in line with the antioxidant activities of the leaf extracts (PE, LE, and PLE) and pod extracts (PdEs) identified through the DPPH assay in this study. Since the antioxidant properties TPC and TFC were the average values among three locations of the short- or tall-stem phenotypes, large deviations in IC_50_ values could be seen, especially in the DPPH radical scavenging effects of PdE from the two phenotypes. This indicated the diverse amounts of bioactive compounds in raw plant materials from various sources, determined according to the environmental growth conditions. The similarity between the tall- and short-stem phenotypes based on TPCs, TFCs, and antioxidant properties was confirmed by the PCA of all phytochemical data and antioxidant activities for six sources of plants, which showed a complete overlap between the tall- and short-stem phenotypes (Figure 5A). The variation in the phytochemicals may have resulted from various factors, including plant genotypes, abiotic stresses, and environmental factors such as the soil and microbes [25].

The contents of baicalin, baicalein, chrysin, and oroxylin A were related to the TPC and TFC, along with the antioxidant activities determined by the DPPH, ABTS, and FRAP assays. It was observed that the short-stem phenotype exhibited stronger antioxidant properties than the tall-stem phenotype. This finding encourages the use of the *O. indicum* short-stem phenotype as a raw plant material because it produces a healthy harvest with lower cost and needs less equipment or machinery.

The complete overlap in the PCA of phytochemical contents among PE, LE, and PLE suggested that they were no different and supported the use of the whole leaf rather than separating it into the leaflet and petiole. The cluster of immature pods distinct from the leaf parts (Figure 5B) exhibited weaker responses in the DPPH and ABTS assays (Figure 4). The differences in the phytochemical contents between the clusters of *O. indicum* pods and leaves might result from the plant cell compartment of flavonoid biosynthesis that occurs in chloroplast-rich organs, such as leaves and petioles [26,27,28].

### 3.3. Anti-Inflammatory Properties of O. indicum S-BK Leaf and Pod Extracts

Leaves and pod extracts of *O. indicum* S-BK exhibited inhibitory effects on NO production, which corresponded to the activities of biomarkers. There have been reports that baicalin, baicalein, chrysin, or oroxylin A possess anti-inflammatory properties. Baicalin and baicalein are potent anti-inflammatory inhibitors through NO inhibition, PGE2 production, reduction in pro-inflammatory cytokines, including TNF-α, IL-1β, and IL-6, and suppression of iNOS, COX-2, and TNF-α at the gene and protein expression levels in LPS-induced RAW264.7 macrophages [29,30]. Chrysin has an immunomodulatory effect by inducing cell proliferation and decreases pro-inflammatory cytokines (TNF-α and IL-6) as well as pro-inflammatory mediators (iNOS and COX-2) in LPS-stimulated RAW 264.7 macrophages [31]. Additionally, oroxylin A inhibits inflammation by suppressing the production of various cytokines through the calcium–STAT pathway [32] and the regulation of Nrf2 signaling [33].

Although baicalin and baicalein exert potent NO-inhibitory activities, the high contents of these compounds in PLE did not lead to stronger potency than LE. This result might indicate the effect of the other potent substances in LE such as scutellarein and its glycosides [34,35,36], biochanin A [37,38], and many of the unidentified compounds. Therefore, evaluation of the potency of activities of the extracts should be undertaken based on biological assays, while the biomarker content is suitable for the quality control or chemical characterization of the plant’s raw materials or extracts.

Due to the comparable effects of LE and PLE on NO inhibition, utilizing the whole leaf for *O. indicum* extract preparation offers several advantages in anti-inflammatory product development. It reduces the time required and means there is no waste of petiole parts through separating leaflets from the whole leaf. This approach also leads to a higher extraction yield and maximizes cost-effectiveness.

## 4. Materials and Methods

### 4.1. Chemicals

All general chemicals and solvents used in this study were of analytical grade. The solvents for the HPLC quantitative analysis were of HPLC grade. Quercetin, DPPH, ABTS, FRAP, N (ω)-nitro-L-arginine methyl ester (L-NAME), sodium nitrite, lipopolysaccharide, and gallic acid were purchased from Sigma-Aldrich (St. Louis, MO, USA). Baicalin, baicalein, chrysin, and oroxylin A were sourced from Biopurify (Chengdu, China), N-(1-Naphthyl) ethylenediamine dihydrochloride (NED) was obtained from PanReac (Germany). Dulbecco’s modified Eagle’s medium (DMEM), penicillin–streptomycin, and fetal bovine serum (FBS) were purchased from Hyclone (Logan, UT, USA), while 0.25% trypsin–EDTA, and trypan blue were from Gibco (New York, NY, USA), and 3-(4,5-dimethylthiazol-2-yl)-2,5-diphenyltetrazolium bromide (MTT) were sourced from Invitrogen (Lane County, OR, USA).

### 4.2. Plant Materials and Extraction

Immature *Oroxylum indicum* pods and leaves were collected from open fields in various locations. The samples of the tall-stem phenotype were collected from plants of heights more than 15 m, naturally grown in Sukhothai, Roi Et, and Buengkarn provinces of Thailand, while those of the short-stem phenotype were approximately 5 m in height and collected from commercial fields in Khon Kaen and Buengkarn province, Thailand.

All samples were washed. The leaf samples were divided into 3 groups—leaflet (L); petiole, rachis, and rachilla (P); and whole leaves (PL)—as shown in Figure 2. The immature pods (Pds) were cut into small pieces. After that, they were dried in a 50 °C hot-air oven and ground. The crude extract of each sample was obtained through the maceration of 150 g dried powder in 80% EtOH 1500 mL (ratio 1:10 *w*/*v*) twice, each time for three days. Then, it was filtered through a Büchner funnel, evaporated using a rotary evaporator, and lyophilized until dry. The crude extracts (LE, PE, PLE, and PdE) were kept at −20 °C until use.

### 4.3. Validation of the HPLC Method and the Quantitative Analysis of Extracts

The HPLC system with an autosampler (Agilent 1260UV, Agilent Technology, Santa Clara, CA, USA) consisted of a photodiode array detector. The column was a VDSpher PUR 100 C18-E (250 × 4.6 mm, 5 µm, Berlin, Germany). The mobile phase consisted of solvent A (0.1% formic acid (*v*/*v*) in H_2_O) and solvent B (0.1% formic acid (*v*/*v*) in acetonitrile). The column was equilibrated with 15%B, and a gradient was applied as follows: 0–60 min: 15–60%B; 60–75 min: 60–100%B; 75–80 min: 100%B; and 80–90 min: 100–85%B. The flow rate was kept at 1 mL/min and the injection volume was 10 µL. The column temperature was maintained at 25 °C. The peaks were monitored at a wavelength 254 nm.

The HPLC validation of baicalin, baicalein, chrysin, and oroxylin A was included in the linearity range, the limit of detection (LOD), the limit of quantification (LOQ), precision, and accuracy, which followed the AOAC guidelines [7].

Standard baicalin, baicalein, chrysin, and oroxylin A were dissolved in MeOH and were diluted into the concentration ranges 5–160, 5–80, 5–640, and 5–160 µg/mL, respectively. The concentration within the linearity range was plotted against the average area under the peak from 5 replicates of the standard substance injection to achieve the linear formula. Correlation coefficients (R^2^) for linearity greater than 0.99 were accepted.

The limit of detection (LOD) and limit of quantification (LOQ) are the lowest concentrations of compounds that can be detected and quantified by HPLC, respectively. They are determined by a signal-to-noise ratio of greater than 3 for the LOD and greater than 10 for the LOQ.

The precision of the method for baicalin, baicalein, chrysin, and oroxylin A was assessed from each standard—baicalin (5, 10, 20, 40, 80, 160 µg/mL), baicalein (5, 10, 20, 40, 80 µg/mL), chrysin (5, 10, 20, 40, 80, 160, 320, 640), and oroxylin A (5, 10, 20, 40, 80, 160 µg/mL)—on the same day (intra-day) and on three different days (inter-day) in five replicates. The peak area of each biomarker was determined. The percentages of relative standard deviation (%RSD) were calculated as follows, which should be lower than 0.2%:

% RSD = (standard deviation/mean) × 100(1)

The accuracy is expressed as the recovery percentage. PLE (1 mg/mL in MeOH) from *O. indicum* T-SK was used. It was spiked with 20, 40, and 80 µg/mL of baicalin, baicalein, chrysin, or oroxylin A in triplicate. Accuracy was assessed by comparing the experimental concentrations with the theoretical concentrations (calculated from the standard graph), which should be within the range of 95–105%. This percentage of recovery was calculated using the following formula:

% Recovery = (experimental concentration/theoretical concentration) × 100(2)

The quantitative analysis of extracts was performed by the validated HPLC method using 10 µL of 1 mg/mL extract. Biomarker contents were calculated from the linear equation of each compound. The results are shown in concentration (µg/mL), percentage of the extract, or percentage of dry weight.

### 4.4. Total Phenolic and Total Flavonoid Contents

The total phenolic content (TPC) was determined using the Folin–Ciocâlteu colorimetric method. The reaction, consisting of 20 μL 1 mg/mL crude extract, 100 μL 10% (*v*/*v*) Folin–Ciocâlteu reagent, and 80 μL 7% (*w*/*v*) Na_2_CO_3_, was performed in a 96-well plate. The mixture was mixed and kept in darkness at room temperature for 30 min. The absorbance was measured at 760 nm using a microplate reader (En-sight, PerkinElmer, Waltham, MA, USA). The calibration curve of gallic acid was generated as Y = 0.0044x + 0.1467, R^2^ = 0.9944. The total phenolic content is presented as milligrams of gallic acid equivalent (GAE) per gram of extract.

The total flavonoid content (TFC) was determined in a 96-well plate. The reaction contained 1 mg/mL crude extract or various concentrations of quercetin at 100 μL, mixed with 5% (*w*/*v*) NaNO_3_ at 20 μL and left for 6 min before adding 10% (*w*/*v*) AlCl_3_ at 35 μL, then incubated in the darkness at room temperature for 30 min. Then, the absorbance was measured at 430 nm using a microplate reader. Quercetin was used to establish the standard graph, which provided the linear formula: y = 0.0152x + 0.0127 (R^2^ = 0.9996). The total flavonoid content of the extract was presented as milligrams of quercetin equivalent (QE) per gram of extract.

### 4.5. Antioxidant Assays

#### 4.5.1. DPPH Radical Scavenging Assay

The extracts were diluted in MeOH at a concentration range of 7.81–1000 μg/mL. The reaction was performed in a 96-well plate consisting of 100 μL sample and 100 μL 0.2 mM DPPH, incubated at room temperature for 30 min and protected from light. Then, the absorbance was measured at 517 nm using a microplate reader. The percentage of radical scavenging was calculated using the following equation:

% DPPH radical scavenging = [(A_DPPH_ − A_sample_)/A_DPPH_] × 100 (3)

where A_DPPH_ was the absorbance of DPPH, A_sample_ was the absorbance of sample, and the IC_50_ (µg/mL) is reported as the concentration of the sample that scavenged 50% of the DPPH free radicals. The positive control Trolox was used.

#### 4.5.2. ABTS Assay

The ABTS radical was prepared by mixing 4.95 mM potassium persulfate and 7 mM ABTS solution and kept in the dark at room temperature for 16 h. The mixture was diluted with MeOH until the absorbance value was 0.7 ± 0.03 at 734 nm. The assay was performed in a 96-well plate by mixing 150 µL of the ABTS radical solution with 50 µL of the extract and incubating for 10 min in the darkness. Then, the reaction was measured for the absorbance at 734 nm. The percentage of radical scavenging was calculated using the following equation:

% ABTS radical scavenging = [(A_ABTS_ − A_sample_)/A_ABTS_] × 100 (4)

where A_ABTS_ was the absorbance of ABTS and A_sample_ was the absorbance of the sample. The results are reported as IC_50_ (µg/mL). Trolox was used as the positive control.

#### 4.5.3. FRAP Assay

FRAP reagent, composed of 300 mM acetate buffer (pH 3.6), 10 mM TPTZ in 40 mM HCl, and 20 mM FeCl_3_ at a ratio of 10:1:1 (*v*/*v*), was prepared. Then, 350 μL of the FRAP reagent was mixed with 10 µL of 1 mg/mL extract and incubated for 15 min at room temperature in darkness. The absorbance was measured at 593 nm using a microplate reader. The calibration curve was established using Trolox in the concentration range of 1–1000 µg/mL. Y = 0.0026x + 0.0419, R^2^ = 0.9886. FRAP values are reported as milligrams of Trolox equivalent per gram of extract.

### 4.6. Inhibition of Nitric Oxide Production in LPS-Induced RAW264.7 Macrophages

RAW 264.7 macrophages were kindly provided by Assistant Professor Dr. Pramote Mahakunakorn, Faculty of Pharmaceutical Sciences, Khon Kaen University, Thailand.

Cell viability assays were performed using the MTT method [39] in a 96-well cell culture plate in the presence and absence of lipopolysaccharide (LPS). Cell viability percentages were calculated with the following equation:

% cell viability = (A_treated_/A_control_) × 100 (5)

where A_treated_ was the absorbance of the treated cells and A_controls_ was the absorbance of the control.

The inhibition of NO production in LPS-induced RAW264.7 macrophages was assayed in a 96-well cell culture plate. Cells were treated with the sample containing 100 ng/mL LPS. The positive control was 250 µM L-NAME in the presence of LPS. The NO production was calculated from measuring of nitrite content in the culture media using Griess reagent. NO-inhibition percentages were calculated using the following equation:

% NO inhibition = (NO_LPS_ − NO_sample_)/(NO_LPS_ − NO_untreated control_) × 100 (6)

where NO_untreated control_ was the nitrite content of the cell culture medium of the untreated control (without LPS and extract), NO_LPS_ was the nitrite amount in the cell culture medium of the LPS-induced cells, and NO_sample_ was the nitrite content in the cell culture medium of the treated cells in the presence of LPS.

### 4.7. Statistical Analysis

The data were analyzed using independent-sample *t*-tests to compare the two groups, and one-way analysis of variance (ANOVA) was applied for comparisons within the groups, with values presented as means ± standard deviation (SD). The comparison of data between tall- and short-stem phenotypes is expressed as means ± standard error of the mean (SEM). Statistical analysis was performed in SPSS version 23.0 software (IBM Corp., New York, NY, USA). Principal component analysis (PCA) was carried out using R version 4.4.1 for Windows (Posit Software, Boston, MA, USA)

## 5. Conclusions

Variable chemical compositions and antioxidant activities were found among the sources of *O. indicum*, which did not relate to the plant stem height phenotypes. The results from this study support the utilization of leaf and pod of the tall- and short-stem phenotypes of this plant. Hence, if the cost of the harvesting process is considered, short-stem phenotypes should be selected. The different chemical contents between the leaf extract (both whole leaf and the separated part) and pod extract resulted in different anti-inflammatory effects. The extracts from the whole leaf and leaflet demonstrated better anti-inflammatory activities than the pods. As the entire leaf extract (PLE) showed a close strength in NO inhibition to that of the leaflet extract (LE) and was more potent than the petiole, rachis, and rachilla extract (PE), we advise the use of the whole leaf for further development as an anti-inflammatory product. Due to the variations in chemical profiles and biological activities among the plant locations of *O. indicum*, quality control of the raw materials is necessary. This includes quantitative analysis of biomarker contents and bioactivity testing of the extracts to ensure they meet the criteria for product preparation.

## Figures and Tables

**Table 1:** Physical appearance of the leaflet and pod of Indian trumpet tree *O. indicum* in Thailand.

Phenotype	Province	Code	Leaflet	Immature Pod
**Tall stem**	Buengkarn	T-BK	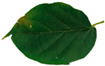	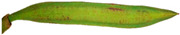
	Sukhothai	T-SK	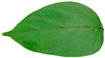	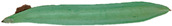
	Roi-Et	T-RE	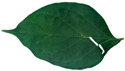	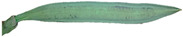
**Short stem**	Buengkarn	S-BK	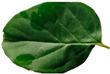	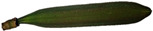
	Khon Kaen (Urban)	S-KK1	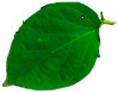	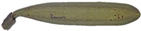
	Khon Kaen (Rural)	S-KK2	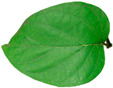	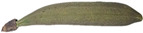

**Table 2:** Yield percentage, total phenolic and total flavonoid contents of *O. indicum* extracts from tall- and short-stem phenotypes.

	Leaves						Immature Pods	
	PE		LE		PLE		PdE	
	Tall	Short	Tall	Short	Tall	Short	Tall	Short
**% Yield**	8.99 ± 0.61	8.14 ± 0.90	15.56 ± 2.21	14.24 ± 2.68	14.53 ± 1.24	12.51 ± 2.13	30.75 ± 3.28	25.35 ± 4.35
**TPC** **(mg GAE/g extract)**	53.34 ± 5.37	69.68 ± 15.33	89.18 ± 5.14	82.18 ± 7.41	89.18 ± 5.14	82.18 ± 7.41	17.36 ± 2.17	21.03 ± 5.42
**TFC** **(mg QE/g extract)**	15.39 ± 1.39	20.18 ± 4.79	38.30 ± 2.58	45.87 ± 6.96	28.07 ± 3.76	43.94 ± 10.53	5.04 ± 0.65	3.39 ± 0.48

Data expressed as mean ± standard error of mean (SEM), *n* = 3.

**Table 3:** Comparison of baicalin, baicalein, chrysin, and oroxylin A levels in the leaf extracts (PEs, LEs, and PLEs) and the immature pod extract (PdE) of *O. indicum* tall- and short-stem phenotypes.

Biomarkers	Leaves (% Extract *w/w*)						Immature Pods (% Extract *w/w*)	
	Petiole and Rachis Extracts (PEs)		Leaflet Extracts (LEs)		Whole-Leaf Extracts (PLEs)		Pod Extracts (PdEs)	
	Tall	Short	Tall	Short	Tall	Short	Tall	Short
**Baicalin**	2.68 ± 0.57	2.72 ± 0.55	2.42 ± 0.54	1.50 ± 0.13	1.90 ± 0.28	1.94 ± 0.18	2.36 ± 0.39	2.37 ± 0.67
**Baicalein**	1.98 ± 0.35	2.18 ± 0.18	2.2 ± 0.21	1.08 ± 0.18	1.82 ± 0.25	2.75 ± 0.82	0.89 ± 0.15	2.57 ± 0.23
**Chrysin**	ND	ND	0.67 ± 0.11	ND	ND	0.52 ± 0.24	ND	ND
**Oroxylin A**	1.10 ± 0.20	1.01 ± 0.22	2.64 ± 0.56	1.63 ± 0.24	1.56 ± 0.42	2.41 ± 0.72	ND	ND

Data expressed as mean ± standard error of mean (SEM), *n* = 3; ND means the amount could not be calculated as the area under the peak was outside the linearity range of the standard graph.

**Table 4:** IC_50_ values of the extracts and biomarkers, along with the biomarker contents of *O. indicum* S-BK.

Samples	IC_50_ (µg/mL)	Content * (% *w/w* Extract)			
		Baicalin	Baicalein	Chrysin	Oroxylin A
**PE**	235.33	0.73 ± 0.00	2.03 ± 0.05	0.61 ± 0.02	1.86 ± 0.04
**LE**	57.61	2.67 ± 0.02	1.83 ± 0.01	0.55 ± 0.02	2.57 ± 0.05
**PLE**	77.58	5.30 ± 0.03	6.03 ± 0.12	1.50 ± 0.02	5.28 ± 0.05
**PdE**	303.01	0.70 ± 0.00	1.65 ± 0.00	ND	ND
**Baicalin**	5.86	-	-	-	-
**Baicalein**	7.31	-	-	-	-
**Chrysin**	>400	-	-	-	-
**Oroxylin A**	10.46	-	-	-	-

* The content was determined from HPLC analysis of the extract at a concentration of 1 mg/mL. ND meant the amount could not be calculated as the area under the peak was outside the linearity range of the standard graph.

## Data Availability

The original contributions presented in the study are included in the article/Supplementary Material, further inquiries can be directed to the corresponding author/s.

## Supplementary Materials

The following supporting information can be downloaded at https://www.mdpi.com/article/10.3390/plants13152110/s1. Figure S1: Inhibition of NO production on LPS-activated RAW264.7 macrophages; Table S1: Yield percentage and chemical contents of *O. indicum* leaf and pod extracts from 6 locations; Table S2: Method validation of biomarkers in *O. indicum* extracts according to the AOAC guidelines; Table S3: Antioxidant activities of leaf extracts (PE, LE, PLE) and pod extracts (PdE) of the individual *O. indicum* source.
