# Supplementary material for: A Comparative Study of Two Oroxylum indicum (L.) Kurz. Phenotypes Based on Phytochemicals and Antioxidant Effects, and the Anti-Inflammatory Activity of Leaf and Pod Extracts
Source: Plants (Basel). 2024 Jul 30;13(15):2110. doi: 10.3390/plants13152110 (PMC11314318; doi:10.3390/plants13152110)
Supplement: Supplementary file 1 [file plants-13-02110-s001.zip › plants-3072615-supplementary.pdf]

Supplementary Materials

A Comparative Study of two *Oroxylum indicum* (L.) Kurz. Phenotypes Based on Phytochemicals and Antioxidant Effects, and the Anti-inflammatory Activity of Leaf and Pod Extracts

Pattaraphorn Panomai, Suthasinee Thapphasaraphong and Natsajee Nualkaew*

Faculty of Pharmaceutical Sciences, Khon Kaen University, Khon Kaen 40002, Thailand;
pattar@kkumail.com (P.P); sutpit1@kku.ac.th (S.T.)
*Correspondence: nnatsa@kku.ac.th; Tel. +66-43-202-178

Supplementary Tables

Table S1. Yield percentage and chemical contents of *O. indicum* leaf and pod extracts from 6 locations

**Plant**	**Part**	**Extract**	**% yield**	**Total chemical contents***		**Biomarker contents (% w/w extract)***			
				TPC (mg GAE/g extract)	TFC (mg QE/g extract)	Baicalin	Baicalein	Chrysin	Oroxylin A
**T-BK**	Leaf	PE	8.8	32.94 ± 3.69	11.73 ± 0.42	0.72 ± 0.00	2.03 ± 0.05	0.61 ± 0.02	1.86 ± 0.04
		LE	16.0	71.96 ± 1.32	28.57 ± 1.78	2.52 ± 0.01	1.81 ± 0.02	0.55 ± 0.02	2.57 ± 0.05
		PLE	16.0	62.87 ± 3.48	15.50 ± 0.20	2.52 ± 0.01	6.04 ± 0.12	1.50 ± 0.02	5.28 ± 0.05
	Pod	PdE	37.32	18.32 ± 1.51	3.52 ± 0.17	0.75 ± 0.00	1.65 ± 0.00	ND	ND
**T-SK**	Leaf	PE	8.0	58.40 ± 2.11	13.63 ± 0.69	0.77 ± 0.00	2.81 ± 0.27	ND	ND
		LE	19.2	88.17 ± 2.33	40.87 ± 2.09	2.03 ± 0.01	0.92 ± 0.01	ND	1.32 ± 0.02
		PLE	15.5	65.44 ± 3.30	29.14 ± 1.27	0.89 ± 0.07	1.16 ± 0.03	ND	1.11 ± 0.01
	Pod	PdE	27.7	9.92 ± 3.19	4.10 ± 0.04	ND	2.83 ± 0.02	ND	ND
**T-RE**	Leaf	PE	10.1	68.70 ± 2.19	20.83 ± 0.18	1.05 ± 0.01	1.70 ± 0.03	ND	0.82 ± 0.01
		LE	11.5	107.42 ± 0.69	45.48 ± 1.78	3.79 ± 0.02	ND	ND	1.01 ± 0.01
		PLE	12.1	96.66 ± 2.85	41.46 ± 1.68	5.16 ± 0.02	1.05 ± 0.12	ND	0.83 ± 0.01
	Pod	PdE	27.3	23.86 ± 3.08	7.51 ± 1.29	0.64 ± 0.00	3.21 ± 0.03	ND	ND
**S-BK**	Leaf	PE	6.3	46.28 ± 1.25	39.18 ± 1.09	0.73 ± 0.00	1.36 ± 0.01	ND	0.49 ± 0.00
		LE	18.8	69.46 ± 0.35	45.74 ± 1.35	2.67 ± 0.02	1.68 ± 0.07	ND	0.92 ± 0.01
		PLE	16.5	51.89 ± 0.23	85.04 ± 3.07	5.30 ± 0.03	1.10 ± 0.03	ND	ND
	Pod	PdE	42.1	42.42 ± 1.60	1.48 ± 0.29	0.70 ± 0.00	0.34 ± 0.00	ND	ND
**S-KK1**	Leaf	PE	9.2	130.37 ± 6.19	12.74 ± 0.52	0.68 ± 0.00	3.38 ± 0.01	0.36 ± 0.01	1.84 ± 0.02
		LE	14.4	111.43 ± 3.01	70.02 ± 2.71	1.05 ± 0.03	1.92 ± 0.01	0.76 ± 0.01	2.23 ± 0.02
		PLE	11.7	203.02 ± 4.42	31.14 ± 1.81	1.66 ± 0.00	2.80 ± 0.00	1.08 ± 0.02	3.22 ± 0.05
	Pod	PdE	21.4	8.93 ± 4.61	4.43 ± 0.17	0.73 ± 0.00	0.98 ± 0.02	ND	ND
**S-KK2**	Leaf	PE	8.9	32.42 ± 1.37	8.64 ± 0.15	0.78 ± 0.03	1.20 ± 0.02	ND	0.96 ± 0.01
		LE	9.5	65.67 ± 5.65	21.88 ± 0.41	0.75 ± 0.01	3.01 ± 0.14	1.00 ± 0.02	4.76 ± 0.06
		PLE	9.3	81.05 ± 2.84	15.65 ± 0.25	0.74 ± 0.00	1.56 ± 0.02	ND	1.06 ± 0.01
	Pod	PdE	12.6	11.74 ± 1.51	4.27 ± 0.04	0.83 ± 0.00	1.34 ± 0.02	ND	ND

*Data are expressed as the mean ± standard error, n = 3.

ND meant the amount could not be calculated as the area under the peak was outside the linearity range of the standard graph

Table S2. Method validation of biomarkers in *O. indicum* extracts according to the AOAC guidelines

Parameters	Baicalin	Baicalein	Chrysin	Oroxylin A	Acceptance criteria
Linearity range (µg/mL)	5 – 160	5 – 80	5 – 640	5 – 160	-
Linear equation	y = 13.369x - 64.562	y = 33.761x - 52.642	y = 46.967x + 50.702	y = 36.903x - 17.302	-
R^2	0.9996	0.9997	0.9991	0.9995	≥ 0.990
LOD (ng)	25	10	5	10	-
LOQ (ng)	50	25	10	50	-
Intra-day (% RSD)	0.14 – 1.86	0.05 – 1.53	0.12 – 1.16	0.15 – 1.41	< 2
Inter-day (% RSD)	0.23 – 1.84	0.14 – 1.19	0.17 – 1.77	0.16 – 0.92	< 2
% Recovery	92.43 – 102.93	96.76 – 101.72	95.45 – 100.76	99.98 - 104.90	90 - 107

Table S3. Antioxidant activities of leaf extracts (PE, LE, PLE) and pod extracts (PdE) of the individual *O. indicum* source.

Plant	Part	Extract	Antioxidant		
			IC_{50} DPPH (μg/mL)	IC_{50} ABTS (μg/mL)	FRAP (mg TEAC/g extract)
**T-BK**	Leaf	PE	93.31 ± 3.39	30.75 ± 0.90	66.13 ± 6.19
		LE	57.61 ± 1.50	15.04 ± 0.19	179.67 ± 9.92
		PLE	63.90 ± 5.36	23.91 ± 0.21	240.10 ± 33.23
	Pod	PdE	249.64 ± 0.87	59.75 ± 5.53	80.11 ± 4.29
**T-SK**	Leaf	PE	46.46 ± 0.54	39.56 ± 1.27	116.42 ± 3.66
		LE	20.31 ± 0.35	23.39 ± 5.32	220.85 ± 20.88
		PLE	26.54 ± 2.16	16.62 ± 0.40	225.47 ± 26.71
	Pod	PdE	61.59 ± 1.12	104.04 ± 1.96	60.99 ± 1.56
**T-RE**	Leaf	PE	43.16 ± 1.06	16.60 ± 0.59	200.97 ± 35.60
		LE	19.85 ± 0.35	17.41 ± 0.77	161.96 ± 28.49
		PLE	25.94 ± 2.05	16.80 ± 0.40	190.45 ± 32.52
	Pod	PdE	55.52 ± 3.22	60.77 ± 5.72	80.88 ± 13.04
**S-BK**	Leaf	PE	31.38 ± 0.57	26.93 ± 0.80	104.87 ± 10.70
		LE	28.55 ± 1.02	17.38 ± 0.36	197.50 ± 4.07
		PLE	12.45 ± 0.26	8.97 ± 0.06	279.10 ± 7.86
	Pod	PdE	286.60 ± 5.36	18.31 ± 0.44	89.60 ± 1.60
**S-KK1**	Leaf	PE	36.81 ± 3.86	18.04 ± 0.23	149.78 ± 3.91
		LE	31.48 ± 3.86	22.32 ± 0.54	130.92 ± 6.94
		PLE	19.51 ± 1.26	18.16 ± 6.87	244.84 ± 28.00
	Pod	PdE	87.66 ± 2.42	21.00 ± 0.42	148.62 ± 25.83
**S-KK2**	Leaf	PE	56.38 ± 0.58	16.52 ± 0.31	145.41 ± 12.44
		LE	48.37 ± 0.81	23.29 ± 0.69	153.62 ± 20.62
		PLE	59.32 ± 0.34	18.08 ± 6.45	121.93 ± 8.16
	Pod	PdE	65.14 ± 1.47	27.59 ± 0.71	147.08 ± 16.00

Data are expressed as the mean ± standard error.

The IC_{50} values of Trolox are 5.91 ± 0.02, and 4.18±0.05 μg/mL from the DPPH and ABTS assays, respectively.

Supplementary Figures

Figure S1. Inhibition of NO production on LPS-activated RAW264.7 macrophages.

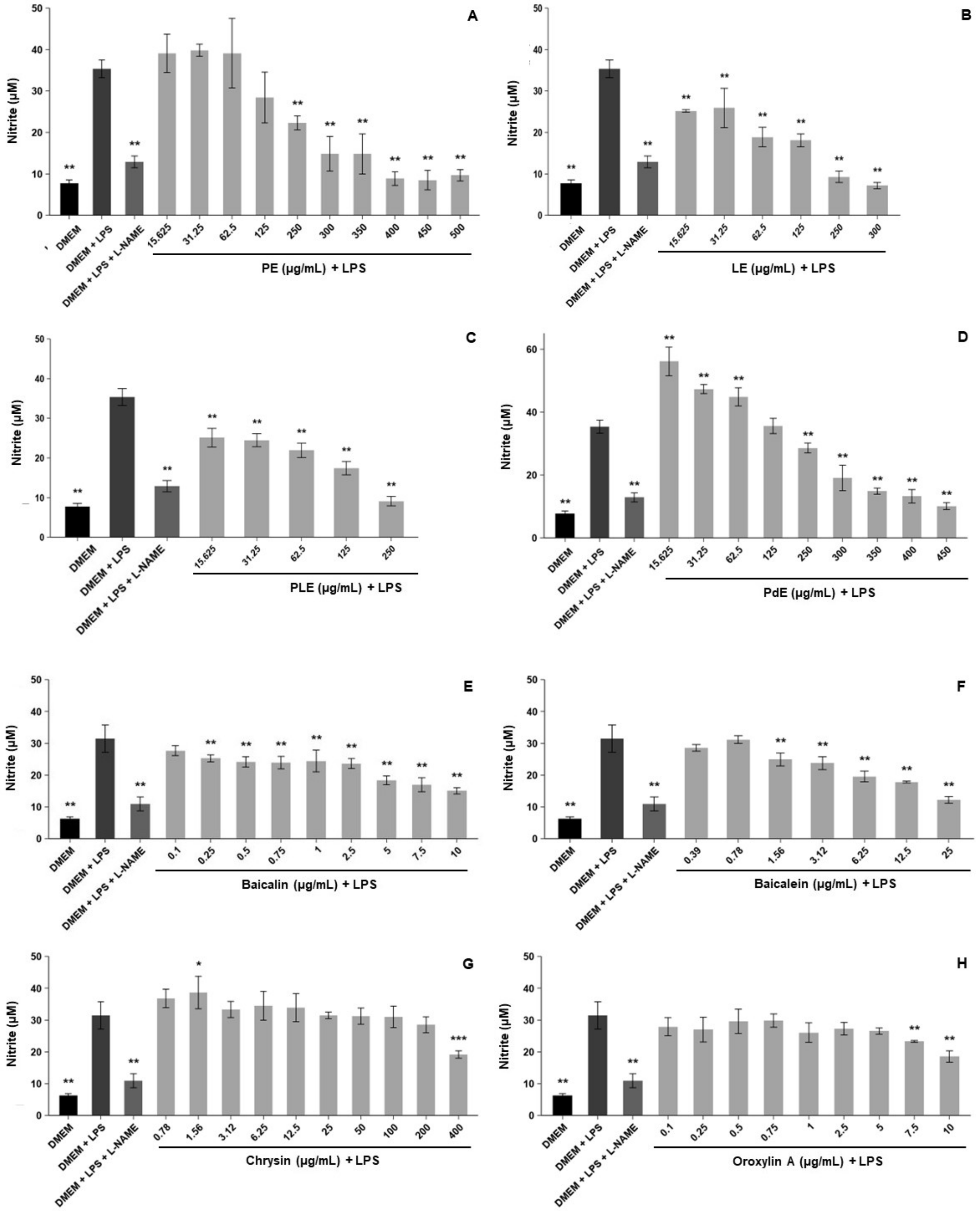

Figure S1. Inhibition of NO production on LPS-activated RAW264.7 macrophages: **(A-D)**: *O. indicum* S-BK extracts (LE, PE, PLE, and PdE, repectively); **(E-H):** biomarkers (baicalin, baicalein, chrysin, and oroxylin A, respectively). L-NAME was used as a positive control. The nitrite contents in the cell culture medium was shown. Data are expressed as the mean ± SD. *$p < 0.05$, compared with the DMEM+100 ng/mL LPS, $n = 4$. The positive control was 250 µM L-NAME + 100 ng/mL LPS.
